# *Triatoma rosai* sp. nov. (Hemiptera, Triatominae): A New Species of Argentinian Chagas Disease Vector Described Based on Integrative Taxonomy

**DOI:** 10.3390/insects11120830

**Published:** 2020-11-26

**Authors:** Kaio Cesar Chaboli Alevi, Jader de Oliveira, Ariane Cristina Caris Garcia, Daniel Cesaretto Cristal, Luiza Maria Grzyb Delgado, Isadora de Freitas Bittinelli, Yago Visinho dos Reis, Amanda Ravazi, Ana Beatriz Bortolozo de Oliveira, Cleber Galvão, Maria Tercília Vilela de Azeredo-Oliveira, Fernanda Fernandez Madeira

**Affiliations:** 1 Laboratório de Parasitologia, Faculdade de Ciências Farmacêuticas, Universidade Estadual Paulista “Júlio de Mesquita Filho” (UNESP), Rodovia Araraquara-Jaú km 1, 14801-902 Araraquara, SP, Brazil; kaiochaboli@hotmail.com (K.C.C.A.); jdr.oliveira@hotmail.com (J.d.O.); daniel.cristal@hotmail.com (D.C.C.); 2 Instituto de Biociências de Botucatu, Universidade Estadual Paulista “Júlio de Mesquita Filho” (UNESP), Rua Dr. Antônio Celso Wagner Zanin, 250, Distrito de Rubião Junior, 18618-689 Botucatu, SP, Brazil; ariane.garcia@outlook.com (A.C.C.G.); uiza_grzyb.1998@hotmail.com (L.M.G.D.); isadora_bittinelli@hotmail.com (I.d.F.B.); yagoreis@outlook.com.br (Y.V.d.R.); amandaravazi95@gmail.com (A.R.); 3 Laboratório de Biologia Celular, Instituto de Biociências, Universidade Estadual Paulista “Júlio de Mesquita Filho” (UNESP), Letras e Ciências Exatas, Rua Cristóvão Colombo 2265, 15054-000 São José do Rio Preto, SP, Brazil; anabbortolozo@gmail.com (A.B.B.d.O.); tercilia.vilela@unesp.br (M.T.V.d.A.-O.); fernanda.bio56@hotmail.com (F.F.M.); 4 Laboratório Nacional e Internacional de Referência em Taxonomia de Triatomíneos, Instituto Oswaldo Cruz (FIOCRUZ), Av. Brazil 4365, Pavilhão Rocha Lima, sala 505, 21040-360 Rio de Janeiro, RJ, Brazil

**Keywords:** triatomines, morphology, morphometry, molecular, experimental crosses, Chagas disease

## Abstract

**Simple Summary:**

Although all triatomines are potential vectors of Chagas' disease, there are species with greater or lesser vectorial importance. Therefore, the correct identification of triatomines species is essential for the vector control programs. In general, triatomines are identified by external morphological characters. However, some species are very similar or even morphologically identical, being important the use of complementary analyses for the correct identification of species. For this reason, this study focused on the use of morphological, morphometric, molecular data, and experimental crosses to describe *Triatoma rosai* sp. nov., a new species of Argentinian Chagas disease vector. Significant morphological and morphometric differences, associated with phylogenetic support and high mortality rate of the hybrids made it possible to confirm the specific status of *T. rosai* sp. nov., emphasizing the importance of integrative analyses for the taxonomy of triatomines.

**Abstract:**

Chagas disease is the most prevalent neglected tropical disease in the Americas and makes an important contribution to morbidity and mortality rates in countries where it is endemic since 30 to 40% of patients develop cardiac diseases, gastrointestinal disorders, or both. In this paper, a new species of the genus *Triatoma* is described based on specimens collected in the Department San Miguel, Province of Corrientes, Argentina. *Triatoma rosai* sp. nov. is closely related to *T. sordida* (Stål, 1859), and was characterized based on integrative taxonomy using morphological, morphometric, molecular data, and experimental crosses. These analyses, combined with data from the literature (cytogenetics, electrophoresis pattern, molecular analyses, cuticular hydrocarbons pattern, geometric morphometry, cycle, and average time of life as well as geographic distribution) confirm the specific status of *T. rosai* sp. nov. Natural *Trypanosoma cruzi* infection, coupled with its presence mostly in peridomestic habitats, indicates that this species can be considered as an important Chagas disease vector from Argentina.

## 1. Introduction

Chagas disease is the most prevalent neglected tropical disease in the Americas [1] and makes an important contribution to morbidity and mortality rates in countries where it is endemic [2,3] since 30 to 40% of patients develop cardiac diseases, gastrointestinal disorders, or both [2]. It is estimated that 8 million people worldwide are infected by *Trypanosoma cruzi* (Chagas, 1909) (Kinetoplastida, Trypanosomatidae) (etiologic agent of Chagas disease) [3] and about 70 million people are living in areas with contamination risk [4], causing incapacity in infected individuals and more than 10,000 deaths per year [3,4].

Although there are several forms of contamination—organ transplantation of an infected donor, laboratory accidents, ingestion of contaminated food or liquids (sugarcane, acai, and raw meat), congenital (vertically between mother and child), or can happen through blood transfusion [5]—vectorial transmission by triatomines (Hemiptera, Triatominae) is the most important from an epidemiological point of view [3]. Thus, vector control remains the most useful method to prevent infection [3].

Currently, there are 154 species of triatomines described, grouped into 18 genera and five tribes [6]. Although there are species with a greater or lesser degree of importance in the transmission of Chagas disease, all triatomines, of both sexes and at any stage of development after hatching, are considered as potential vectors of this disease described more than 110 years ago [7,8].

*Triatoma sordida* (Stål, 1859) (Hemiptera, Triatominae) is an endemic species in South America, distributed in Brazil, Argentina, Bolivia, Paraguay, and Uruguay, in the Atlantic forest, Caatinga, Cerrado, Chaco, and Pantanal biomes [9,10,11]. The taxonomic history of this species has been discussed since the 1960s when Abalos and Wygodzinsky [12] observed morphological variation among *T. sordida* populations from Santiago del Estero (northeastern Argentina). Subsequently, Actis et al. [13] evaluated the electrophoretic profiles of hemolymph proteins among Argentinean sylvatic samples with domestic specimens from Brazil and significant differences were observed. However, these wild populations of *T. sordida* from Argentina were described as *T. garciabesi* Carcavallo et al., 1967 [14]. After 12 years, this species was synonymized with *T. sordida* [8] and based on differences in their morphology, isoenzymes, and chromosomal characteristics were once again validated as *T. garciabesi* [15]. Noireau et al. [16] also observed a high genetic distance between *T. sordida* from Bolivia and considered the occurrence of the phenomenon of cryptic speciation for the first time. However, Panzera et al. [17] showed that one of the populations used by Noireau et al. [16] was *T. garciabesi* and not *T. sordida*.

Although these cases of “intraspecific variation” (which were interspecific [17]) have been performed incorrectly, intraspecific polymorphisms have been reported among populations of *T. sordida* from Brazil, Argentina, and Bolivia: Panzera et al. [17,18] based on chromosomal (constitutive heterochromatin pattern and FISH with a 45S rDNA probe) and molecular data (mitochondrial gene COI) observed differences between these populations and suggested the existence of at least two possible different species associated with *T. sordida* (*T sordida sensu stricto*), namely *T. sordida* Argentina and *T. sordida* La Paz (differences confirmed by the cuticular hydrocarbons pattern [19]). Morphometric analyses also differentiated *T. sordida* from Argentina and the specimens from Brazil and Bolivia and emphasized the importance of additional studies to confirm the specific validity of the new taxon(s) [20].

Thus, based on several analyses based on integrative taxonomy (morphological, morphometric, molecular data and experimental crosses), as well as information from the literature (cytogenetics, electrophoresis pattern, molecular analyses, cuticular hydrocarbons pattern, geometric morphometry, cycle and average time of life as well as geographic distribution) that support the specific status of *T. sordida* from Argentina as a new taxon, we describe *Triatoma rosai* sp. nov.

## 2. Materials and Methods

### 2.1. Sampling

We examined specimens from Department San Miguel, Province of Corrientes, Argentina, characterized initially as *T. sordida* using the taxonomic key for the genus *Triatoma* Laporte, 1832 published by Lent and Wygodzinsky [8]. All studied species were kept alive at the Triatominae Insectarium of the São Paulo State University “Julio de Mesquita Filho”, School of Pharmaceutical Sciences, Araraquara, São Paulo, Brazil.

### 2.2. Morphological Identification and Description

The description of *T. rosai* sp. nov. included 7 males and 7 females previously characterized as *T. sordida* (based on the dichotomous key by Lent and Wygodzinsky [8]). *Triatoma rosai* sp. nov. was compared with 9 male and 9 female specimens of *T. sordida* from Riacho dos Machados, Minas Gerais, Brazil (MG/Brazil) deposited in the Dr. Jose Maria Soares Barata Triatominae Collection (CTJMSB) of the São Paulo State University “Julio de Mesquita Filho”, School of Pharmaceutical Sciences, Araraquara, São Paulo, Brazil.

### 2.3. Morphological Studies in Scanning Electron Microscopy

For the comparative morphological study, 2 males and 2 females of *T. rosai* sp. nov. and 2 males and 2 females of *T. sordida* (MG/Brazil) from Araraquara Triatominae Colony (CTA) were used. For morphological characterization of the triatomines in Scanning Electron Microscope (SEM) (according to Rosa et al. [21]), four individuals of each locality were used, emphasizing the study of the pronotum, scutellum, as well as female external genitalia. For this study, the insects were cleaned in ultrasonic devices, dehydrated in graded series of alcohol, oven-dried at 45 °C for 20 min, and then fixed in small aluminum cylinders with colorless enamel. Afterward, they were metalized by sputtering for two minutes with 10 mA of power. After the metallization process, the samples were analyzed and photographed on the Topcon SM-300 SEM (Digital, Hasunuma-cho, Tokyo, Japan).

### 2.4. Morphological Studies in Optical Microscopy

For the comparative morphological study of *T. rosai* sp. nov. and *T. sordida* we used images of the male's head, pronotum, and genitalia. For optical microscopy (OM), adult specimens of *T. rosai* sp. nov. (3 males and 3 females) and of *T. sordida* (MG/Brazil) (3 males and 3 females) were used. The analyses were performed using the Leica MZ APO Stereoscope Microscope (Digital, Wetzlar, Germany) with the Motic Images Advanced 3.2 plus image analysis system coupled. 

### 2.5. Morphometric Studies 

For measurement analyses, adult specimens of *T. rosai* sp. nov. (7 males and 7 females), and *T. sordida* (MG/Brazil) (9 males and 9 females) were used. The measurements of the head, thorax, abdomen, pronotum, and scutellum were performed using the Leica MZ APO Stereoscope Microscope with the Motic Images Advanced 3.2 plus image analysis system coupled. The data obtained were organized in the Excel 2007^®^ spreadsheet software and presented as a table. The measurements of the thorax and abdomen parameters were statistically analyzed in the GraphPad Prism program version 7 for Windows, using Welch’s *t*-test.

### 2.6. Molecular Phylogeny and Genetic Distance

Five specimens of *T. sordida* (*T. sordida sensu stricto*) (Brazil: Posse/Goiás and Seabra/Bahia), *T. sordida* La Paz (Bolivia: La Paz), and *T. rosai* sp. nov., were used for molecular analyses. For DNA extraction, using the DNeasy Blood and Tissue kit (QIAGEN^®^, Hilden, Germany), one or two legs were placed in a 1.5 mL microtube, dipped into liquid nitrogen, and ground to powder. A 561-bp fragment of the mitochondrial (mt) cytochrome b gene (cyt b) and a 798-pb fragment of the nuclear Internal Transcribed Spacer-1 gene (ITS-1) were polymerase chain reaction (PCR) amplified as described in Monteiro et al. [22] and Tartarotti and Ceron [23], respectively. Amplicons were purified using a GFX PCR DNA & Gel Band kit (GE Healthcare and Life Technology^®^, Chicago, IL, USA) and both strands were subjected to Sanger sequencing reactions (ABI Prism^®^ BigDye^®^ Terminator v.3.1 Cycle Sequencing Kit, Applied Biosystems, Foster City, CA, USA) and run on an ABI 3730 sequencer. The sequences of all individuals were analyzed by using BioEdit Alignment Editor 7.0.5.3 [24] and a consensus sequence was obtained for each DNA segment. Sequences from other Brazilian localities (MG and MS), *T. garciabesi*, *T. guasayana* Wygodzinsky and Abalos, 1949 and *Panstrongylus megistus* (Burmeister, 1835) available on GenBank were also added to the alignment (Table 1). The sequences were aligned using the ClustalW Multiple Alignment tool [25]. Using the MEGA software (Molecular Evolutionary Genetics Analysis) v.6 [26] we constructed a genetic distance matrix using the Kimura-2 parameter distance model for estimation of evolutionary divergence between sequences of the cyt b gene.

For the Bayesian inference, we combined unpublished and GenBank sequences (Table 1). All sequences used were submitted to the MEGA X program [27] and aligned by the Muscle method [28]. The alignments of each molecular marker (COI, Cyt b, 28S, 16S, and ITS-1) were concatenated by name using Seaview 4 software [29], posteriorly being converted on Mesquite program [30]. The data were partitioned for each marker, with the best-fit models of nucleotide substitution (lowest Akaike Information Criterion value) for the phylogenetic reconstruction determined by jModelTest 2 program [31], for each of them: HKY + G for 16S, HKY + I + G for Cyt b, HKY for ITS-1, GTR for 28S, and GTR + G for COI. A Bayesian phylogenetic tree for the complete data set of concatenated sequences was inferred in MrBayes 3.2 [32]. The trees were sampled every 1,000 generations, for a total of 100 million generations, in two independent runs and with a burn-in of 25%. The reliability of clustering patterns in trees was assessed by 1000 bootstrap replicates [33]. Tracer v. 7.0 [34] was used to verify the stabilization moment (ESS values above 200) of the tree samples. The generated tree was visualized and edited in FigTree v.1.4.3 program [35].

### 2.7. Experimental Crosses

Reciprocal experimental crosses were conducted between *T. rosai* sp. nov. and *T. sordida* to evaluate the pre- and/or post-zygotic reproductive isolation barriers [36,37]. The experimental crosses were conducted in the Triatominae Insectary of the School of Pharmaceutical Sciences, according to the experiments of Costa et al. [38] and Mendonça et al. [39], the insects were sexed as 5th instar-nymphs, and males and females were kept separately until they reached the adult stage to guarantee the virginity of the insects used in the crosses. For the experimental crosses, three couples from each set were placed in plastic jars (diameter 5 cm × height 10 cm) and kept at room temperature. Furthermore, intraspecific crosses were also performed for group control. The eggs were collected weekly throughout the female's oviposition periods and the egg fertility rate was calculated. Besides, after the hybrids hatched, the development of 1st instar-nymphs until adults was also monitored weekly to assess the mortality rate.

### 2.8. Distribution Map

Based on Lent and Wygodzynsky [8], Panzera et al. [17,18], Dujardin et al. [40]; Galvão et al. [41], Calderón-Fernández and Juárez [19], and Nattero et al. [20], we prepared a distribution map of *T. rosai* sp. nov. across Argentina provinces. The map was developed in the QGIS 3.12 program [42], using the layers available at the National Geographic Institute [43] having the World Geodetic System 1984 (WGS 84) Coordinate Reference System as a base.

## 3. Results

### 3.1. Systematics

Family Reduviidae Latreille, 1807

Subfamily Triatominae Jeannel, 1919

Genus *Triatoma* Laporte, 1832

*Triatoma rosai* Alevi, Oliveira, Garcia, Cristal, Delgado, Bittinelli, Reis, Ravazi, Oliveira, Galvão, Azeredo-Oliveira, Madeira, 2020 sp. nov. (Figure 1A,B, Figure 2A,B, Figure 3A,C, Figure 4A,C,E, Figure 5A,C,E, Figure 6A,B, Figure 7A–C, Figure 8A,B, Figure 9A–C)

#### Type Material

Holotype: male, ARGENTINA, Department San Miguel, Province of Corrientes, 09.08.1999, collection manual, leg. (CEJMSB).

Paratypes: 9 females and 8 males, ARGENTINA, Department San Miguel, Province of Corrientes, 09.08.1999, collection manual, leg. (CEJMSB).

Additional paratypes. Collection of Triatomines of the Oswaldo Cruz Institute (CTIOC/FIOCRUZ), Rio de Janeiro, Brazil (1 male and 1 female), and Entomological Reference Collection of the Faculty of Public Health (FSP/USP), São Paulo, Brazil (1 male and 1 female).

### 3.2. Description

#### 3.2.1. Male

**Figure 1:**
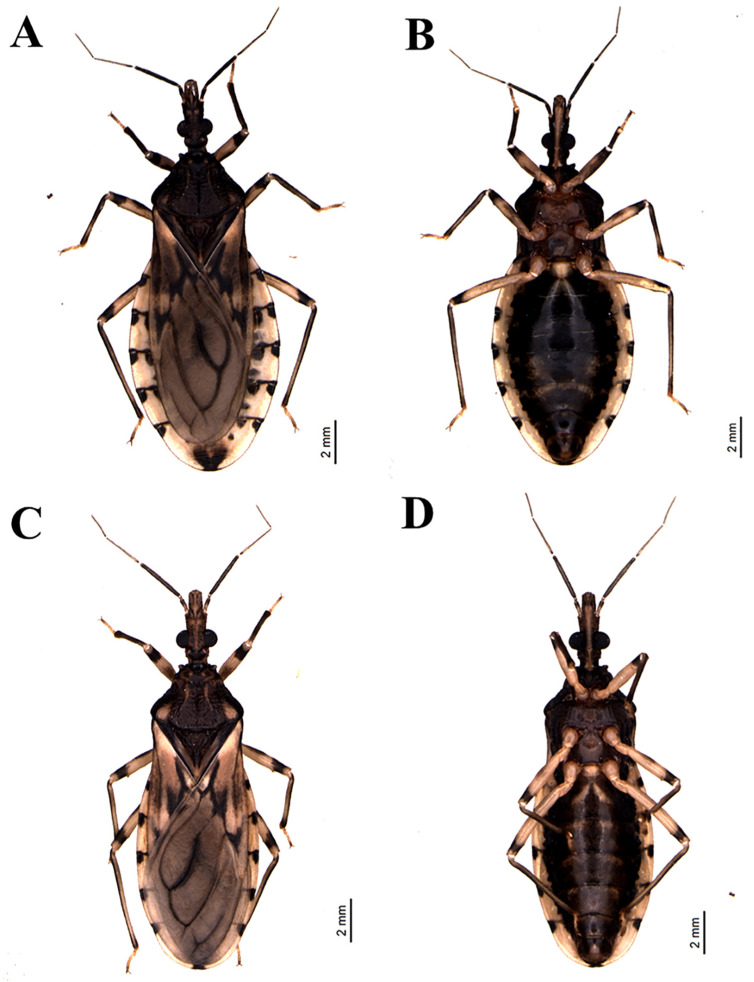
*Triatoma rosai* sp. nov. male (**A**) dorsal view, (**B**) ventral view, *Triatoma sordida* male (**C**) dorsal view, (**D**) ventral view.

**Figure 2:**
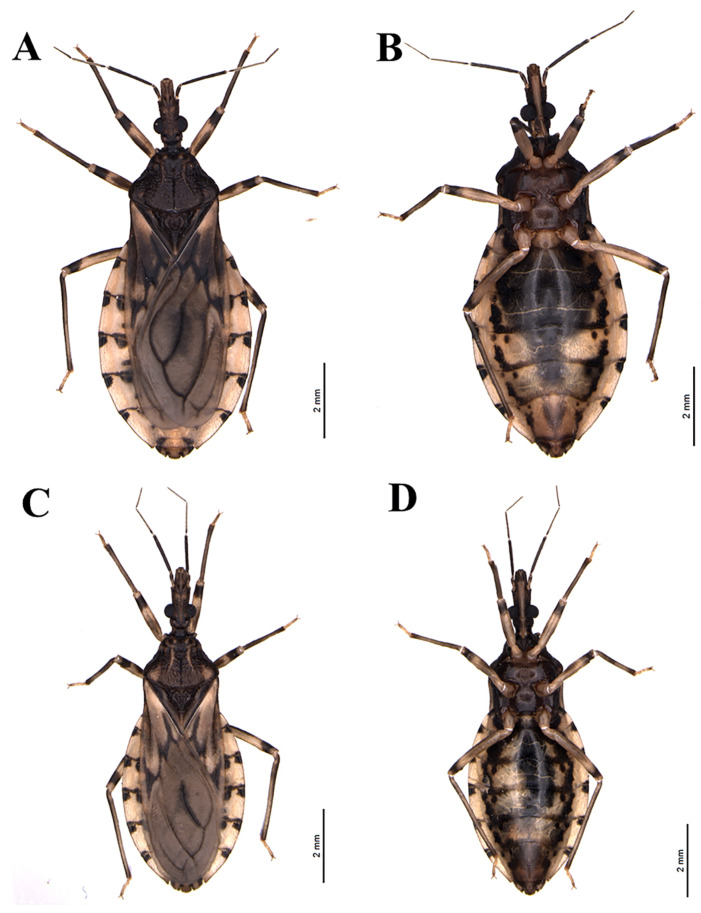
*Triatoma rosai* sp. nov. female (**A**) dorsal view, (**B**) ventral view, *T. sordida* female (**C**) dorsal view, (**D**) ventral view.

**Figure 3:**
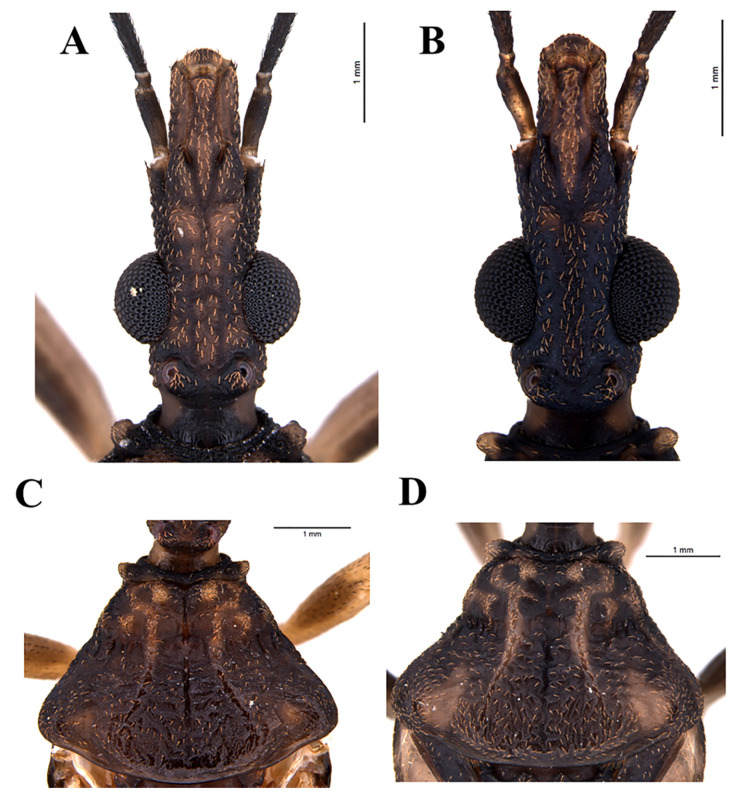
Detail of Head: (**A**) *T. rosai* sp. nov. dorsal view, (**B**) *T. sordida* dorsal view; detail of Pronotum: (**C**) *T. rosai* sp. nov. dorsal view, (**D**) *T. sordida* dorsal view.

Coloring: general brown color with pale yellowish tones with portions or spots darkened to brownish or blackish (Figure 1A,B and Figure 2A,B). Head, including neck, mainly dark brown with two lateral spots in the catch and darkened or blackened ventrally and laterally behind the eyes; ocellar tubercle, the median portion in the space between the eyes and the apices of dark anteniferous tubercles; first and second antennal segments brown and third and fourth brownish; mostly pale lip, with darkened portions (Figure 3A). Labium with the three visible light brown segments (Figure 1B and Figure 2B). Thorax mainly dark brown in general; pronotum brown (Figure 3C); anterolateral angles blackened with yellowish ends; longitudinal medial groove in the anterior lobe, variable in length, darkened, a spot in pairs in the anterior lobe of yellowish color, posterior lobe of brown coloration; dark brown scutellum on the sides and brownish in the central portion with yellowish apex. Legs generally light brown to yellowish with the following portions or markings darkened: coxae completely yellowed; anterior and medium femurs with an ill-defined complete median or submedian black ring and the apex slightly darkened; these markings can be variable or absent; posterior femur with a distal submedian ring slightly large and incomplete ventrally; tibiae with brownish sub-basal rings, larger in the posterior tibiae and slightly subapically darkened or with the apical third or darkened apical quarter; yellowish tarsi (Figure 1A,B and Figure 2A,B). Hemelytra mostly grayish; with yellowish spots at the base of the dorsal surface, laterally, and in the lateral portion, basally; two black spots at the basal angle of the second disk cell of the membrane; sometimes the basal portions of the veins in that cell are also black. Abdomen, in dorsal view: brownish tergites with some slightly darkened portions, as an intersegmental bridge between the first and second segments, except their lateral portions (Figure 1A and Figure 2A). Connexion with distal areas having two dark spots and whitish pale yellowish basal part, each spot occupying approximately 1/4 of the respective segment; a medium-large oval black spot in the sternite VIII, in ventral view: sternites mostly yellowish; blackened sternites, except in the lateral portions; a pair of wavy black spots on the side, which in some specimens is smaller, incomplete, stained with a pale color in its center, or divided into a pair of sub-median spots, or joined to the sideband by the darkened posterior portion of the segment ( 1B and 2B) The markings on the ventral connected segments are generally the same as those on the dorsal portions, but more variable, weakly marked or partially or completely absent in some segments.

**Figure 4:**
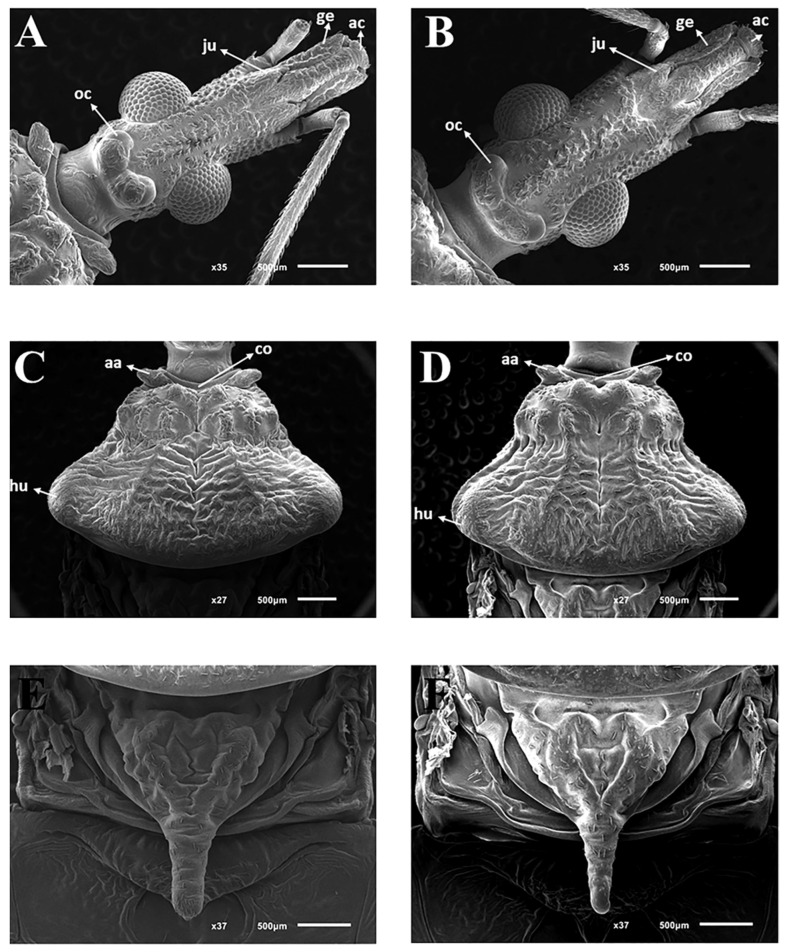
Detail of Head by SEM: (**A**) *T. rosai* sp. nov. by dorsal view, (**B**) *T. sordida* by dorsal view; Detail of Pronotum by SEM: (**C**) *T. rosai* sp. nov. dorsal view (**D**) *T. sordida* dorsal view; Detail of scutellum by SEM: (**E**) *T. rosai* sp. nov. dorsal view (**F**) *T. sordida* dorsal view. ac: anteclypeus; ge: gena; ju: jugum; oc: ocellus; aa: anterolateral angle; co: collar; hu: humeral angles.

**Figure 5:**
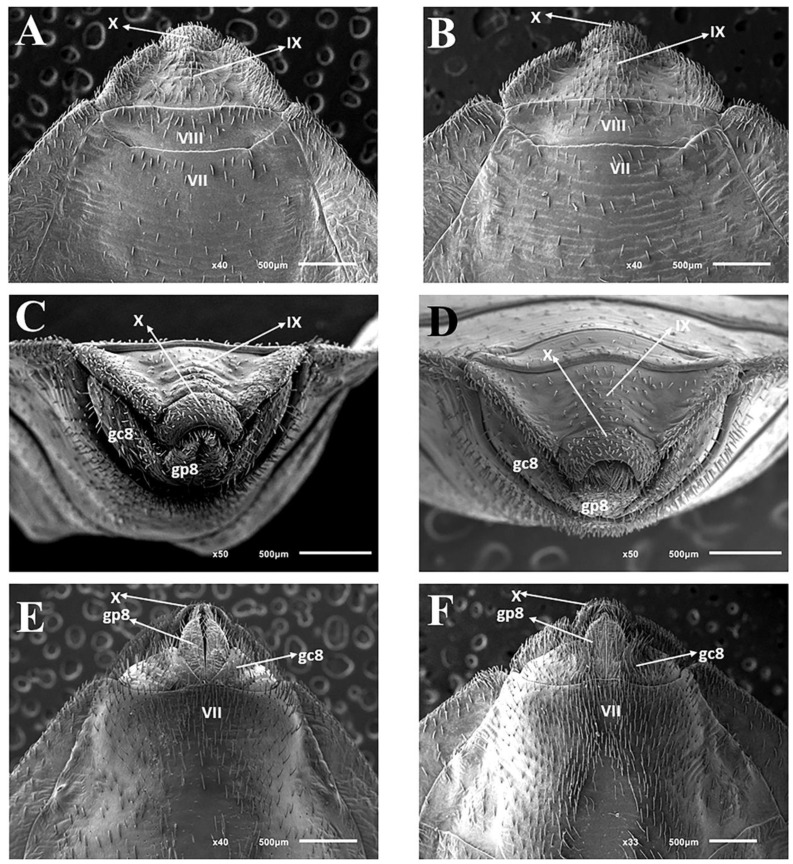
Female external genitalia by SEM: *T. rosai* sp. nov. (**A**) dorsal view, (**C**) posterior view, (**E**) ventral view, *T. sordida*: (**B**) dorsal view, (**D**) posterior view, (**F**) ventral view. Gc 8: gonocoxite; Gc 9: gonapophyse; Gp 8: gonapophyse, VII, VIII, IX: tergites; X: segment.

Vestiture: when recorded in SEM, it is possible to perceive that the general integument is covered by short, thin, somewhat curved bristles, for example Figure 4A and Figure 5A,C,E. Besides, the following vestiture characteristics, also evident under the optical stereomicroscope, were registered: head: smooth eyes; sparse short or slightly longer bristles, clear to yellowish in the distal portion of the clypeus, numerous on the lip; some bristles scattered in the anterior and lateral portions of the base of the first visible lip segment; second visible lip segment with some short, erect bristles inserted on the lateral and subapical surfaces; last lip segment with many upright, long, thin and clear scattered setae.

Clypeus narrow slightly enlarged behind the middle. Genae tapered and slightly apically pointed, distinctly extending beyond the level of the apex of the clypeus. Jugae, apically subangular. Eyes in lateral view reaching the lower level, but not reaching the level of the superior surface of the head. Anteniferous tubercles are inserted behind the middle of the anteocular region. The first antennal segment below the middle of the clypeus level. The first rostral segment is very short, not extending exactly to the level of the apex of the yokes, the second reaching the level of the posterior margin of the eyes (Figure 1A,B, Figure 2A,B, Figure 3A, Figure 4A).

Anterior lobe of pronotum with discal and lateral tubercles. Submedian carinae extending near the posterior border of the pronotum. Humeral angles are slightly curved, slightly angular. Anterolateral projections of the pronotum small, but detached, narrowly apically rounded. Scutellum has a well-developed central depression. Large scutellar process, about three-quarters of the length of the main body of the scutellum, horizontal, subcylindrical, slightly tapering apically, obliquely truncated distally in the lateral view. Hemelytra approaching or reaching the apex of the seventh urotergite, in males and females I reached half of the seventh urotergite (Figure 1A, Figure 2A, Figure 3C, Figure 4C).

Relatively slender legs, anterior femurs longer than broad. The anterior and middle femurs with subapically robust pair of denticles. Spongy fossulae in the anterior and middle tibias of males, absent in females. Venter convex, weakly flattened in some specimens of both sexes, delicately striated across, sparsely defined. Spiracles variable in position, from adjacent to the connective internal margin to distant from the internal margin by twice its diameter (Figure 1A,B, Figure 2A,B).

**Figure 6:**
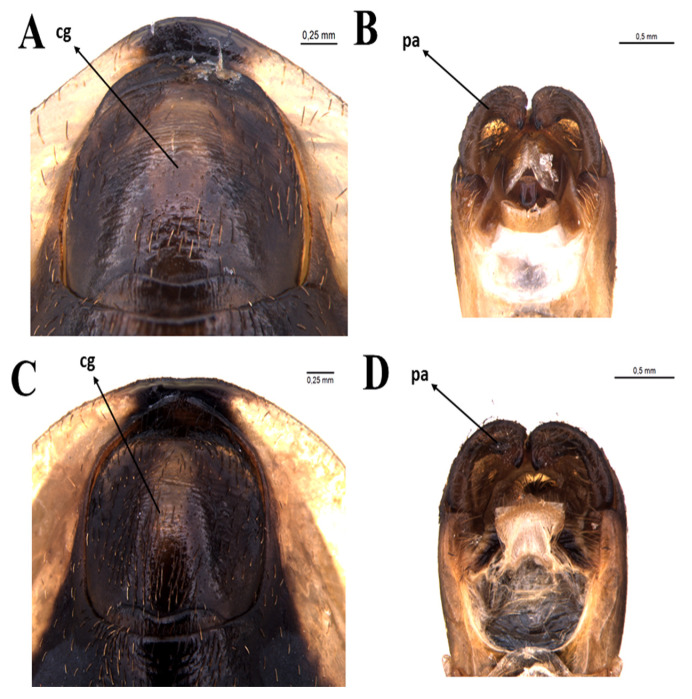
Detail of genital capsule *T. rosai* sp. nov.: (**A**) dorsal view, (**B**) ventral view, *T. sordida*: (**C**) dorsal view, (**D**) ventral view. cg: genital capsule, pa: paramere.

**Figure 7:**
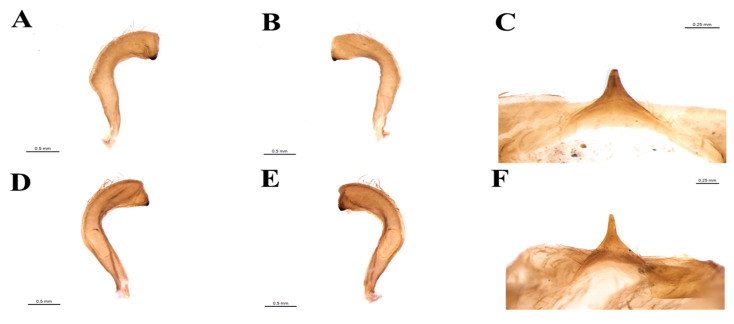
Paramere of *T. rosai* sp. nov.: (**A**) dorsal view, (**B**) ventral view; (**C**) median process of the pygophore; Paramere of *T. sordida*: (**D**) dorsal view, (**E**) ventral view; (**F**) median process of the pygophore.

**Figure 8:**
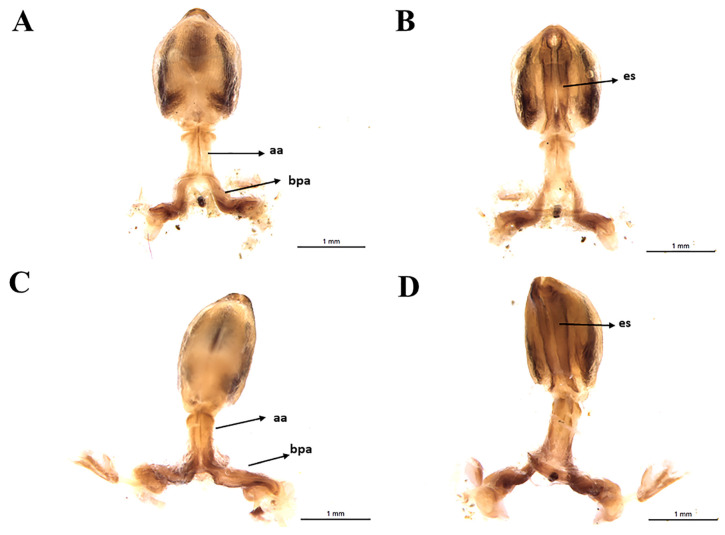
Phallus of *T. rosai* sp. nov.: (**A**) dorsal view, (**B**) ventral view, *T. sordida*: (**C**) dorsal view, (**D**) ventral view. bpa: basal plate arm, aa: articulatory apparatus, es: endosomal struts.

**Figure 9:**
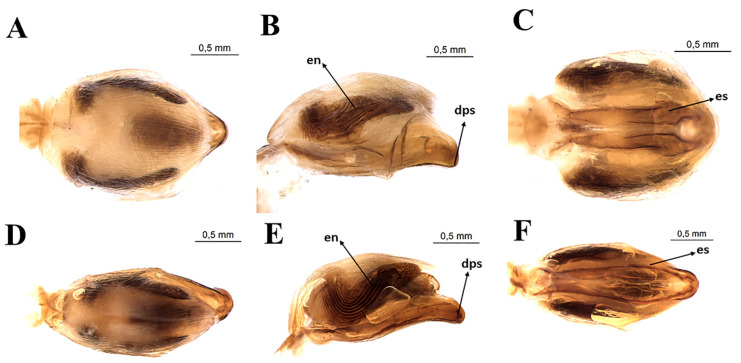
Phallus of *T. rosai* sp. nov.: (**A**) dorsal view, (**B**) lateral view, (**C**) ventral view; *T. sordida*: (**D**) dorsal view, (**E**) Lateral view, (**F**) ventral view. en: endosoma, dps: dorsal phallothecal sclerite, es: endosoma struts.

Male genitalia: genital capsule rounded, in the ventral view, does not show the exposure of the pigophore (Figure 6A,B) is smooth and shiny integument; in dorsal view (Figure 6A). Sclerotized, triangular (equilateral) pigophore median process (Figure 7C), slightly larger towards the apex; apical margin almost straight, slightly curved (Figure 7C). Parameters (pa) slightly not exposed when the genital capsule is in situ (Figure 6A) or in a ventral view (Figure 6B); their apexes almost touch each other in a resting position (Figure 6B). Symmetrical, elongated, curved approximately in the middle third; slightly larger in the apical quarter; truncated apex, with short subapical tooth on the lower margin; mainly glabrous, with some thin bristles scattered and a group of short, thicker bristles mediated to the subapical tooth (Figure 7A,B), the basal plate arm with moderately short and not curved basal plate arms (bpa), connected by a narrow basal plate bridge (Figure 8A). Endosomal arms formed by a pair of parallel arms, almost straight in the middle portion, larger in the basal portion, joined at the base and at the apex, which are continuous with the dorsal fusion of sclerite-sclerite endosome (Figure 9A). After inflation, the endosome takes on an irregular shape with rough streaks (Figure 9B). The endosome wall striated longitudinally in the basal portion in the ventral view (Figure 9B).

#### 3.2.2. Female

Similar to males in general.

### 3.3. Female External Genitalia

Dorsal view of female external genitalia: lines (1 + 1) limiting the seventh segment with the connexivum are regular (Figure 5A). Posterior portions (1 + 1) of the seventh segment at the limit with the eighth segment and connecting end in rounded ends (Figure 5A). Connexiva (1 + 1) of the seventh segment ends in a pointed tip (Figure 5A). The eighth segment shows a trapezoidal shape with rounded edges (Figure 5A). The ninth segment is wavy and the side portions (1 + 1) form raised flaps (Figure 5A). The posterior portions (1 + 1) of the ninth segment have the shape of rounded tips that take the approximate shape of the letter W (Figure 5A).

Posterior view of female external genitalia: the central part of the region that divides the eighth and ninth segments is straight; the junction of the ninth segment IX with the central portion is convex. Being elevated, the lateral portions (1 + 1) form depressions in the anteroposterior direction (Figure 5B). The boundaries between the ninth and tenth segments are opened on the sides and closed in the central area (Figure 5B). The tenth segment is semicircular (Figure 5B). Gonocoxites 8 are penniform, while gonapophyses 8 are almost triangular (Figure 5B).

Ventral view of female external genitalia: the limit of the seventh segment with gonocoxites 8 and gonapophyses 8 is concave on the sides (1 + 1) and convex in the central portion (Figure 5C). Posterior portions of the seventh segment connective end in rounded ends (Figure 5C). From this point of view, gonocoxites 8 and gonapophyses 8 (1 + 1) are triangular (Figure 5C).

### 3.4. Measurement Data

The measurement data of the studied characters were made available in a table, the characters were: total body length, head length, the internal distance between the eyes, post-ocular head width, pronotum, scutellum, thorax, and abdomen (Table 2).

### 3.5. Distribution

**Figure 10:**
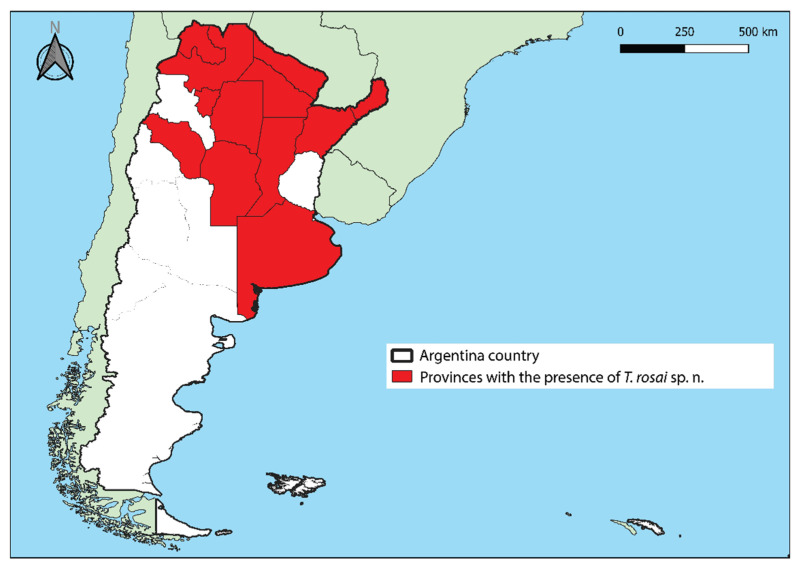
The occurrence of *T. rosai* sp. nov. in Argentina provinces. Made with QGIS.

Based on the article by Panzera et al. [17] which indicates that *T. sordida sensu stricto* and *T. sordida* La Paz were not notified in Argentina; a distribution map was developed from the notifications of *T. rosai* sp. nov. (pointed out in the articles as *T. sordida* from Argentina or *T. sordida* Argentina [8,17,18,19,20,40,41]) in 12 Argentina provinces: Misiones, Corrientes, Santa Fe, Formosa, Chaco, Buenos Aires, Córdoba, Jujuy, Salta, Santiago del Estero, Tucumán and La Rioja (Figure 10).

### 3.6. Observation

The possible existence of this new species was first noted by Panzera et al. [18] and later mentioned by Dujardin et al. [40], Calderón-Fernández and Juárez [19], Panzera et al. [17] and Nattero et al. [20].

### 3.7. Etymology

This species is dedicated to Prof. Dr. João Aristeu da Rosa [Full professor of the School of Pharmaceutical Sciences of São Paulo State University (FCFAR/UNESP), Araraquara, São Paulo, Brazil], one of the world’s top authority on triatomine morphology, who has contributed for more than 30 years with taxonomic and systematic studies of these vectors.

### 3.8. Phylogenetic Reconstruction and Genetic Distances

**Figure 11:**
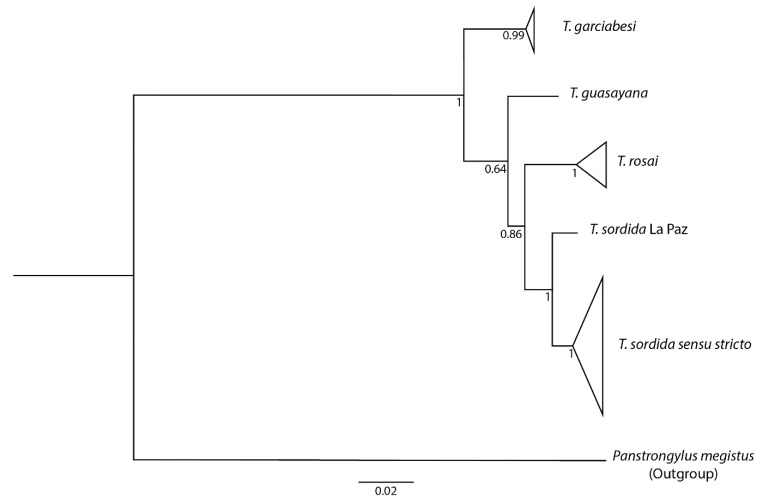
The tree obtained by Bayesian Inference for the concatenated genes COI, cyt b, 28S, 16S and ITS-1. The numbers in the nodes represent the bootstrap values.

Phylogenetic analyses recovered *T. rosai* sp. nov. as an independent lineage of *T. sordida sensu stricto* and *T. sordida* La Paz, as well as *T. garciabesi* and *T. guasayana*, and related it as a sister taxon to *T. sordida* (Figure 11). Pairwise Kimura 2-parameter genetic distances for cyt b gene revealed that *T. rosai* sp. nov. diverges at least 8.5% from the lineages of *T. sordida sensu stricto* (Table 3).

### 3.9. Experimental Crosses

The crosses between *T. rosai* sp. nov. and *T. sordida sensu stricto* resulted in hybrids (absence of pre-zygotic barrier) (Table 4). However, hybrids showed a high mortality rate when compared to parental crosses (post-zygotic barrier by hybrid inviability) (Table 4).

### 3.10. Taxonomic Notes

*Triatoma sordida* (Stål, 1859) (Figure 1C,D, Figure 2C,D, Figure 3B,D, Figure 4B,D,F, Figure 5B,D,F, Figure 6C,D, Figure 7D–F, Figure 8C,D, Figure 9D–F, Figure 12A,B).

Conorhinus sordidus Stål, 1859,

Triatoma sordida Chagas, 1912

Eutriatoma sordida Pinto, 1931

*Triatoma sordida* Lent and Wygodzinsky 1979

#### 3.10.1. List of Synonymous

List of Synonymous: *Conorhinus sordidus* Stål, 1859 and *Eutriatoma Sordida* Pinto, 1931 [11].

#### 3.10.2. Distribution

Distribution: Brazil: Acre, Bahia, Goiás, Maranhão, Mato Grosso, Mato Grosso do Sul, Minas Gerais, Paraná, Pernambuco, Piauí, Rio Grande do Sul, Santa Catarina, São Paulo and Tocantins. Distribution outside Brazil: Bolívia, Paraguay and Uruguay [11,44].

#### 3.10.3. Type Material

**Figure 12:**
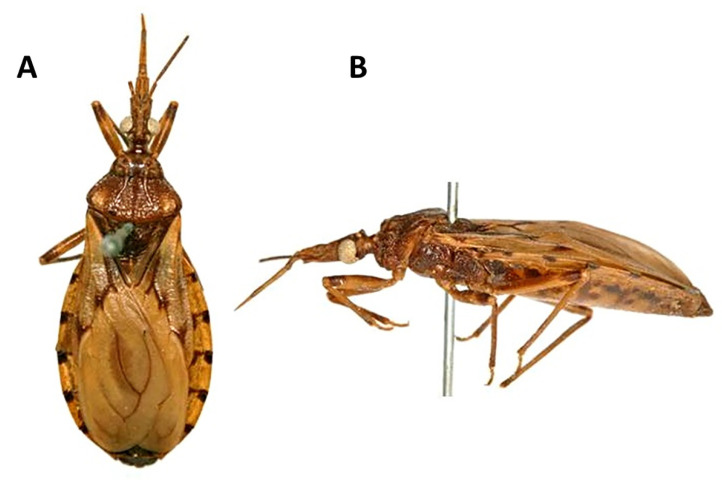
Holotype of *T. sordida* described by Stål in 1859 (as *Conorhinus sordidus*) and deposited in the Zoologisches Museum, Berlim, Alemanha, A—Dorsal view; B—Lateral view.

Type material: deposited at the Zoologisches Museum, Berlin, Germany [45] (Figure 12). 

#### 3.10.4. Comments

Comments: all aspects used in this description of *T. rosai* sp. nov. were compared with the type specimen and with a population from the same state.

### 3.11. Literature Data

#### 3.11.1. Genetics Differences 

##### Cytogenetics Differences

The cytogenetic characterization of *T. rosai* sp. nov. was carried out by Panzera et al. [17,18] and Bardella et al. [46] (termed as *T. sordida* Argentina) and demonstrated great differences when compared to *T. sordida sensu stricto*: *T. rosai* sp. nov. [absence of heterochromatin and CMA_3_/DAPI bands in autosomes, chromocenter formed only by sex chromosomes, Y sex chromosome is DAPI^+^ with CMA_3_^+^ dots, and 45S rDNA clusters were observed in both sex chromosomes (X and Y)] and *T. sordida sensu stricto* [presence of heterochromatic blocks on a chromosomal end (8 to 10 autosomes), all bivalents showed CMA^+^/DAPI^+^ regions co-localized, chromocenter formed by sex chromosomes and autosomes, Y sex chromosome DAPI^+^ and negative for CMA_3_, and 45S rDNA clusters were observed in X sex chromosome].

##### Electrophoresis Pattern

The electrophoretic profile of *T. rosai* sp. nov. (termed as *T. sordida* Argentina) was characterized by Panzera et al. [18]. The authors noted that of the 14 enzyme systems studied, three diagnostic loci evaluated among *T. rosai* sp. nov. and *T. sordida sensu stricto* showed no common alleles [GPI (11), PEP-1 (11), PEP-2 (11), and GPI (33), PEP-1 (22), PEP-2 (22), respectively].

##### Molecular Analyses

Garcia et al. [47], using mtDNA fragments of 12S and 16S ribosomal RNA genes observed, for the first time, phylogenetic divergences between *T. sordida* from Brazil and *T. rosai* sp. nov. (termed as *T. sordida* from Argentina) since the specimens from Argentina were closer to *T. matogrossensis* Leite and Barbosa, 1953 than to *T. sordida* from Brazil. Posteriorly, the phylogenetic analyses performed by Panzera et al. [17] with the mitochondrial gene COI demonstrated that *T. rosai* sp. nov. (termed as *T. sordida* Argentina) constituted a monophyletic clade with 88% bootstrap. The authors also observed a genetic distance between *T. rosai* sp. nov. and *T. sordida sensu stricto* of 5.3%. Belintani et al. [48] performed a phylogenetic study combining COI, cyt b, 16S, 18S and 28S genes and observed that *T. sordida* populations were separated into two well-defined strains: *T. sordida* (Bolivia and Brazil) and *T. rosai* sp. nov. (termed as *T. sordida* Argentina). This division is corroborated by phylogenetic reconstruction, species delimitations based on Poisson Tree Process (PTP), and values of genetic distances. Finally, Madeira et al. [49], through phylogenetic analyzes with cyt b and ND1 genes, clearly demonstrated the difference between *T. rosai* sp. nov. (termed as *T. sordida* from Argentina) and *T. sordida sensu stricto* from different locations in Brazil (Bahia: Gentio de Ouro, Macaúbas, Seabra, and Tanhaçu; Goiás: Posse; Minas Gerais: Brasília de Minas, Catuti, Monte Azul, Porteirinha, and Riacho dos Machados; Mato Grosso do Sul: Corumbá and Pantanal).

#### 3.11.2. Morphological Differences

##### Cuticular Hydrocarbons Pattern

The cuticular hydrocarbons analyses of *T. rosai* sp. nov. (termed as *T. sordida* of Argentina) and *T. sordida sensu stricto* performed by Calderón-Fernández and Juárez [19] comprised that both species showed significantly different hydrocarbon profiles.

##### Geometric Morphometry

Morphometric analyses of the head, wings, and thorax performed in *T. rosai* sp. nov. (termed as *T. sordida* Argentina) and *T. sordida sensu stricto* by Nattero et al. [20] demonstrated divergences between all measured characters. Neighbor-joining trees derived from Mahalanobis distances from head and wing measurements clearly differentiated *T. rosai* sp. nov. and *T. sordida sensu stricto* [20].

#### 3.11.3. Life Cycle and Average Time of Development

According to Oscherov et al. [50], *T. rosai* sp. nov. (termed as *T. sordida* from Argentina) has an average development time of all nymph stages of approximately 303 days, while *T. sordida sensu stricto* has an average time of nymph development of approximately 170 days [51]. Oscherov et al. [50] also reported that the life cycle of T*. rosai* sp. nov. is completed in about 10 months, unlike *T. sordida sensu stricto* that completes its cycle in just five months [52].

##### Geographic Distribution

According to Panzera et al. [17], *T. rosai* sp. nov. (termed as *T. sordida* Argentina) is present in Argentina, Bolivia, and Paraguay, and *T. sordida sensu stricto* is present in Brazil, Bolivia, and Paraguay. Although species may overlap in Bolivia and Paraguay, there are no reports of *T. rosai* sp. nov. in Brazil, as well as *T. sordida sensu stricto* in Argentina (the papers already published with *T. sordida* coming from Argentina were performed with *T. rosai* sp. nov. [9,17,18,19,20,40,41,46,47,48,49,50,53,54,55,56,57,58,59,60,61,62,63,64,65,66,67,68,69,70,71,72,73,74,75])

## 4. Discussion

*Triatoma rosai* sp. nov., in conjunction with *T garciabesi, T. jurbergi* Carcavallo, Galvão, and Lent, 1998, *T. matogrossensis*, *T. sordida*, and *T. vandae* Carcavallo, Jurberg, Rocha, Galvão, Noireau, and Lent, 2002 constitute the monophyletic *T. sordida* subcomplex [48,76]. Several authors, based mainly on morphological data and geographic distribution, considered *T. sordida* subcomplex as a group formed by four species (*T. garciabesi*, *T. guasayana*; *T. patagonica* Del Ponte, 1929, and *T. sordida*) [40,77,78]. Genetic studies (cytogenetics and phylogenetics) have shown that, in fact, *T. guasayana* and *T. patagonica* have an evolutionary relationship with the *T. rubrovaria* subcomplex [48,76].

In addition to *T. rosai* sp. nov., there are 16 species reported in Argentina: *P. coreodes* Bergroth, 1911, *P. geniculatus* (Latreille, 1811), *P. guentheri* Berg, 1879, *P. megistus*, *P. rufotuberculatus* (Champion, 1899), *T. breyeri* Del Ponte, 1929, *T. delpontei* Romaña and Abalos, 1947, *T. eratyrusiformis* Del Ponte, 1929, *T. garciabesi*, *T. guasayana*, *T. infestans* (Klug, 1834), *T. limai* Del Ponte, 1929, *T. patagonica*, *T. platensis* Neiva, 1913, *T. rubrofasciata* (De Geer, 1773) and *T. rubrovaria* (Blanchard, 1843) [41]. Taking into account that *T. garciabesi* shows great morphological similarity with *T. sordida* (which even led to a synonymization event) [8,9,17], the difficulty of morphological differentiation of *T.* *guasayana* and *T. sordida* (especially in the nymphal stages) and the fact that the geographic distribution of both species overlaps in northern Argentina [17], we included these taxa in our phylogenetic studies and confirmed that *T. rosai* sp. nov. is a distinct species.

Several phylogenetic studies showed that *T. rosai* sp. nov. (termed as *T. sordida* Argentina) differs from *T. sordida* (*T. sordida sensu stricto* and *T. sordida* La Paz) [17,47,48,49]. Our results based on integrative taxonomy confirmed that the initial divergences reported as “intraspecific variation” [17,18,19,20] represent interspecific differences, being *T. rosai* sp. nov. an independent strain of *T. sordida*. The last two species of *Triatoma* described (*T. mopan* Dorn et al., 2018 and *T. huehuetenanguensis* Lima-Cordón et al., 2019) were also based on integrative taxonomy, which provides greater reliability of the specific status of these new taxa [79,80].

Among the different tools that can support the integrative taxonomy, the performance of experimental crossings and the analyses of pre- and post-zygotic interspecific reproductive barriers are of great importance to assess the specific status of taxa (based on the biological concept of species) [36,37]. The characterization of pre-zygotic barriers that prevent the formation of hybrids is important from a taxonomic point of view since they demonstrate that the species are genetically isolated (with no interspecific gene flow) [36]. However, the high mortality rate observed among hybrids resulting from crosses between *T. rosai* sp. nov. and *T. sordida sensu stricto* demonstrates in turn that, in addition to the physical barriers that possibly prevent the meeting of these insects in nature (because they live in different countries in South America [17]), there are evolutionary events that lead to a low adaptive value of hybrids. There are several examples of triatomine species that do not have pre-zygotic reproductive barriers, but that had the specific status supported by the post-zygotic barriers (inviability, sterility, or hybrid breakdown) that make hybrids unfeasible [39,81,82,83,84,85,86,87,88].

*Triatoma rosai* sp. nov. (referred in the article as *T. sordida* Argentina) is a wild species, k strategist, present between dry and fallen trunks, tree holes, bromeliads, palm trees, in opossum (*Didelphis* spp.) houses and in dry cacti, which was found naturally infected with *T. cruzi* and is considered responsible for the parasite's wild cycle (mainly associated with bird nests). This species has also been reported in rural peridomestic (such as goat corrals), as well as in domiciliary areas (houses, in general, which were 300 to 2700 m away from pigeon nests) [54,57,59,63,64,67,69,70].

A survey carried out on palm trees in the province of Corrientes, Argentina showed that *T. rosai* sp. nov. (termed as *T. sordida* Argentina) has a high rate of infection with *T. cruzi*, since 38.5% of the specimens analyzed were infected – it is believed that prawns and rodents are the reservoirs of the parasite in palm trees [59]. Maffey et al. [72] also suggest that *T. rosai* sp. nov. probably is the vector of *T. cruzi* genotype I (TcI) among opossums and can act as a secondary vector of the TcVI and TcV genotypes in domestic and peridomestic cycles. These data are of great epidemiological importance since the infection rate of *T. rosai* sp. nov. is extremely high when compared to *T. sordida sensu stricto* (rates ranging from 0.5% to 16.2% [89,90,91,92]) and highlights the importance of epidemiological surveillance of this species in Argentina, where has about two to three million people infected (of which approximately 800,000 are chronic heart patients), being 20 million inhabitants living at risk and, above all, nine out of ten new Chagas' disease cases occurring in children in the country [93].

## 5. Conclusions

Based on the above, we describe *T. rosai* sp. nov., highlight the importance of integrative taxonomy for the description of new species of triatomines, and, above all, demonstrate the need for the attention of vector control programs in Argentina for this new species of Chagas disease vector.

## Figures and Tables

**Table 1:** Species, geographic distribution, and molecular markers used in the phylogenetic studies.

Species	Geographic Distribution	Marker				
		COI	Cyt b	28S	16S	ITS-1
*T. sordida*	Seabra, Bahia, Brazil	MH029692	MH054940	MG417085	-	*
*T. sordida*	Posses, Goiás, Brazil	MH029695	*	MH059640	-	-
*T. sordida*	Monte Azul, Minas Gerais, Brazil	MH029693	MH054941	MG417086	-	-
*T. sordida*	Pantanal, Mato Grosso do Sul, Brazil	MH029694	KC249292	KC249210	KC249075	-
*T. sordida*	Apolo, La Paz, Bolívia	MH029696	MH054942	MH059641	-	*
*T. rosai* sp. nov.	San Miguel, Corrientes, Argentina	MH029697	*	MG417088	-	*
*T. rosai* sp. nov.	San Miguel, Corrientes, Argentina	KC249387	KC249295	KC249213	KC249078	-
*T. garciabesi*	Rivadavia, Salta, Argentina	MH029698	MH054943	MG417089	-	-
*T. garciabesi*	Rivadavia, Salta, Argentina	KC249338	KC249249	KC249158	KC249006	-
*T. guasayana*	Santa Cruz, Bolívia	KC249343	KC249252	KC249162	KC249011	-
*P. megistus*	Nova Prata, Rio Grande do Sul, Brazil	KC249308	KC249228	KC249136	KC248970	AM949580

* Sequences obtained in this study.

**Table 2:** Measurement averages and significance of *T. sordida sensu stricto* and *T. rosai* sp. nov. In bold, we present statistically significant measurements.

Sex	Species	Structures							
**Male**		**HL**	**IE**	**PHW**	**PRO**	**SCU**	**THO**	**ABD**	**TL**
	*T. rosai* sp. nov.	5.174	0.951	**1.490**	**4.717**	**3.077**	**6.704**	**15.117**	**26.98**
	*T. sordida*	4.857	0.966	**1.381**	**4.107**	**2.816**	**6.093**	**14.084**	**25.02**
**Female**	*T. rosai* sp. nov.	5.055	**1.003**	1.486	4.429	3.215	6.531	16.418	27.99
	*T. sordida*	5.208	**1.066**	1.510	4.221	3.214	6.459	16.167	27.81

HL: head length; IE: inner distance between eyes; PHW: postocular head width; PRO: pronotum; SCU: scutellum; THO: thorax; ABD: abdomen, TL: total body length; Data in bold: statistically significant measurements.

**Table 3:** Genetic distance based on cyt b mitochondrial gene. In bold we present the Genetic distance between *T. rosai* sp. nov. and *T. sordida sensu stricto*.

Species	(1)	(2)	(3)	(4)	(5)	(6)	(7)	(8)	(9)
(1) Posse GO *									
(2) Seabra BA *	0.007								
(3) Monte Azul MG *	0.007	0.000							
(4) Pantanal MS *	0.007	0.000	0.000						
(5) ***T. rosai*** **sp. nov**.	**0.085**	**0.087**	**0.087**	**0.087**					
(6) *T. garciabesi*	0.082	0.074	0.074	0.074	0.099				
(7) *T. garciabesi*	0.082	0.074	0.074	0.074	0.097	0.000			
(8) *T. guasayana*	0.078	0.074	0.074	0.074	0.069	0.067	0.067		
(9) *P. megistus*	0.245	0.234	0.234	0.234	0.259	0.240	0.241	0.244	

* *T. sordida* sensu stricto; Data in bold: the Genetic distance between T. rosai sp. nov. and T. sordida sensu stricto.

**Table 4:** Experimental crosses performed between *T. sordida sensu stricto* and *T. rosai* sp. nov.

Experimental Crosses	Number of Eggs	Hatching Rate	Number of Nymphs	Mortality Rate
**Interspecific**				
*♀* *T. sordida sensu stricto* x *T. rosai* sp. nov. ♂	241	77%	186	80%
*♀* *T. rosai* sp. nov. x *T. sordida sensu stricto* ♂	237	59%	139	73%
**Intraspecific**				
*♀* *T. sordida sensu stricto* x *T. sordida sensu stricto* ♂	590	73%	429	20%
*♀* *T. rosai* sp. nov. x *T. rosai* sp. nov. ♂	208	61%	125	17%
